# Programmed Death Ligand-1 expression in stage II colon cancer - experiences from a nationwide populationbased cohort

**DOI:** 10.1186/s12885-019-5345-6

**Published:** 2019-02-12

**Authors:** Ann C. Eriksen, Flemming B. Sørensen, Jan Lindebjerg, Henrik Hager, René dePont Christensen, Sanne Kjær-Frifeldt, Torben F. Hansen

**Affiliations:** 1 Danish Colorectal Cancer Center South, Vejle Hospital, Vejle, Denmark; 2 Institute of Regional Health Research, University of Southern Denmark, Odense, Denmark; 3 Department of Clinical Medicine, University Institute of Pathology, Aarhus University Hospital, and , University of Aarhus, Aarhus, Denmark; 4 Danish Colorectal Cancer Group (DCCG), Aarhus N, Denmark; 5 Research Unit of General Practice, University of Southern Denmark, Odense, Denmark; 6 Department of Pathology, Lillebaelt Hospital, Beriderbakken 4, DK-7100 Vejle, Denmark

**Keywords:** Colon cancer stage II, Prognostic markers, Programmed death ligand-1

## Abstract

**Background:**

Patients suffering from high risk stage II colon cancer (CC) may benefit from adjuvant onco-therapy, but additional prognostic markers are needed for better treatment stratification. We investigated the prognostic value of Programmed Death Ligand-1 (PD-L1) in a true population-based cohort of patients with stage II CC.

**Methods:**

PD-L1 expression on tumour cells was evaluated by immunohistochemistry in 572 colon cancers. Whole sections from tumour blocks representing the deepest invasive front of the primary tumour were used for analysis. A cut-off of 5% positivity was used for dichotomizing the data. The prognostic value was investigated in Cox proportional hazard models for recurrence-free survival (RFS) and overall survival (OS).

**Results:**

Overall, 6% of the tumours were classified as high PD-L1. High PD-L1 was related to female gender (*p* = 0.028), high malignancy grade (< 0.001), right side localization (*p* < 0.001) and microsatellite instability (MSI) (p < 0.001). Thirty-one (18%) of the MSI and 4 (1%) of the microsatellite stable tumours were classified as high PD-L1, respectively. PD-L1 expression provided no prognostic value as a single marker. In patients with MSI tumours, high PD-L1 expression had no significant impact regarding OS or RFS.

**Conclusions:**

PD-L1 expression in tumour cells of stage II CC did not provide any prognostic impact, neither in the entire population-based cohort nor in the group of MSI patients. Additional investigations of the immunogenic microenvironment are needed for evaluating the prognostic information in CC.

## Background

Colon cancer is one of the most common cancers in the Western world. About 1/3 have stage II disease, and this group of patients are in general having a good prognosis with a 5-year overall survival (OS) of approximately 70–80% after surgery alone [1]. Current international guidelines (ASMO and ESMO) do not recommend routine adjuvant chemotherapy in stage II CC, but rather that this treatment be limited to patients having a high risk of recurrence, based on an individual evaluation including high-risk markers [2, 3]. However, the currenct high risk factors are questionable [4], and there is a need for additional prognostic markers for better treatment stratification.

In recent years, the tumour microenvironment has been investigated, and the role of the interaction between cancer cells and the immune system in cancer surveillance has been emphasized [5]. Tumour-infiltrating lymphocytes (TILs) are considered as the host’s immune response against solid tumours, and infiltration by activated CD8+ cytotoxic T-lymphocytes is correlated with better survival of colorectal cancer (CRC) patients [6]. Activated lymphocytes expressing Programmed Death Receptor-1 (PD-1) can bind specific to the ligand Programmed Death Ligand-1 (PD-L1), which is expressed on the cell membrane in malignant epithelial tumours, including colorectal adenocarcinomas [7]. This immune-checkpoint is up-regulated in many tumours, and the interaction of PD-L1 on tumour cells with its receptor PD-1 on the activated T-cells induces a down-regulation of the antigen-stimulated lymphocyte proliferation and cytokine production, resulting in an inhibition of the host-immune response [8].

Current results of the prognostic value of PD-L1 in CRC are controversial. Some studies only report on trends towards worse prognosis for high PD-L1 expression [9, 10], while others identify high PD-L1 to be independently associated with worse recurrence free survival (RFS) [11, 12]. In contrast, other studies report no prognostic impact [13, 14], or even a tendency of high expression of PD-L1 to be related to a better prognosis [15]. However, studies vary greatly in methods and the study populations are highly heterogeneous, including different stages of disease, and no studies have previously investigated the expression of PD-L1 in a cohort exclusively of patients with stage II CC.

With this motivation, the aim of the present study was to evaluate the prognostic impact of PD-L1 in a nationwide, population-based cohort of stage II CC.

## Methods

This study is reported in accordance with the REMARK guidelines [16].

### Patient population

The population and sources of data has previously been descreibed in detail [17]. In brief all patients surgically treated for stage II CC in 2002 in Denmark were identified by a search in the nationwide registry administrated by the Danish Colorectal Cancer Group (DCCG) (*N* = 746). Exclusion criteria were as follows: missing tumour block (*N* = 11), insufficient tissue for analyses (*N* = 2), incorrectly staged patients (*N* = 25), treatment with adjuvant chemotherapy (*N* = 26)/radiotherapy (*N* = 1) and death within 90 days after the operation (*N* = 75). Furthermore patients with loco-advanced disease (*N* = 8) and patients diagnosed with another malignancy prior to CC were excluded from the study (*N* = 26), and the final study population comprised 572 patients.

Histopathological data were obtained by microscopic examination and from the national Patobank containing all pathology reports in Denmark. The term “not assessed” was used if the pathological feature was not described. Clinical data were obtained from The National Patient Registry.

Recurrence primarily occurred within the first five years of follow-up and to encompass the majority of recurrences a follow-up period of seven years was selected.

### Samples

Formalin-fixed, paraffin-embedded tissue blocks were collected from the departments of pathology in Denmark. The tissue blocks were stored and transported at room temperature. One tumour block representing the deepest invasive margin, was selected from each patient. Prior to inclusion, all histological slides from each tumour were evaluated by first a trainee and afterwards an experienced pathologist.

### Immunohistochemistry

From the selected tumour blocks serial 4 μm thick tissue sections were cut and mounted on FLEX IHC Microscope Slides (K8020, DAKO, Glostrup, Denmark). One whole tumour section per patient was used for the evaluation of PD-L1 expression. Staining was performed using a Ventana BenchMark ULTRA (Ventana Medical Systems, Tucson, Arizona, USA) automated immunohistochemistry (IHC) slide staining system. Tissue sections were heated and deparaffinised in EZprep (no.950–102, Ventana). Pre-treatment and demasking were carried out using ULTRA CC1 (no. 950–224) and ULTRA CC2 (no. 950–223), and endogenous peroxidase activity was blocked by Optiview Peroxidase Inhibitor (no. 760–700 Ventana). The slides were incubated with a rabbit monoclonal anti-PD-L1 (clone SP263A, no. 790–4905/741–4905 Ventana) for 16 min. This clone was chosen based on a pilot study. For amplification Optiview HQ Universal Linker (no. 760–700, Ventana) and Optiview HRP Multimer (no. 760–700, Ventana) was each used for 8 min.

The primary antibody was visualized using Optiview H_2_O_2_ and DAB (no. 760–700, Ventana), followed by Optiview Copper (no. 760–700, Ventana). Counterstain was done using Hematoxylin II (no. 790–2208, Ventana) and bluing Reagent (no. 760–2037, Ventana). Finally the histological slides were cover slipped with Tissue-Tek PERTEX (Histolab Products AB, Göteborg, Sweden).

Evaluation of microsattellite instability (MSI) was performed using IHC. Tumours displaying loss of one or more of the 4 mismatch repair proteins (MLH1, MSH2, MSH6 and PMS2) were considered as MSI, whereas tumours with intact mismatch repair proteins were considered as microsatellite stable (MSS). Staining of mismatch repair proteins was performed using a DAKO Autostainer Link 48 (DAKO) with monoclonal mouse antibody against MLH1 (Novocastra, Leica, Germany, clone ES05, dilution 1:100, product code NCL-L-MLH1), MSH2 (Novocastra, Leica, clone 25D12, dilution 1:100, product code NCL-L-MSH2), MSH6 (BD Transduction Laboratories, clone 44/MSH6, dilution 1:200, material number 610919), and PMS2 (BD Pharmingen, clone A16–4, dilution 1:500, material number 556415). Tissue sections were incubated for 30 min at room temperature with the primary antibodies diluted in Envision Flex antibody diluent (code S2022 DAKO). The antibody signal was amplified using Envision Flex+ Mouse(Linker) (DAKO) for 20 min. Bound antibodies were detected using Envision FLEX/HRP (DAKO) and visualized by Envision FLEX DAB (DAKO) and chromogene diluted in Envision Flex Substrate Buffer (DAKO). The sections were incubated in 0.5% CuSO_4_ in TBS buffer pH 7.6 for 10 min to enhance the immunohistochemical staining. Sections were counterstained with Meyer’s hematoxylin (Merck, Damstadt, Germany).

### Scoring of PD-L1 expression

Tumour PD-L1 expression was evaluated based on immunostaining of the cell membrane of the epithelial tumour cells. The immunostaining of the stromal cells were not evaluated. Tumour cells were considered positive when any cell membrane staining (partial of complete) was present. Staining intensity was not evaluated and cytoplasmatic immunoreaction was not considered.

**Fig. 1:**
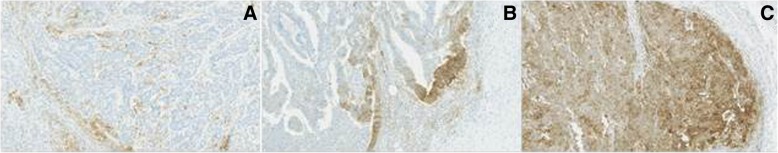
Example of immunohistochemically staining of programmed death ligand-1. **a** ≤ 1% positive tumour cells, **b** 5% < positive tumour cells ≤20%, and **c** > 50% of the tumour cells are positive

The percentage of positive tumour cells were scored semi-quantitatively as 0 (no positive tumour cells), 1 (≤1% positive), 2 (> 1 and ≤ 5% positive), 3 (> 5 and ≤ 20% positive), 4 (> 20 and ≤ 50% positive) and 5 (> 50% positive) (Fig. 1). A subset of 50 randomly selected tumours was examined by a second pathologist in order to assess inter-observer variation. For prognostic evaluation data were dichotomized, using 5% PD-L1 expression as cut-off. In absence of a standardized scoring system the cut-off was based on previously studies [9, 12, 14].

### Statistics

The inter-observer reproducibility of PD-L1 scoring was evaluated by kappa statistics. Simple and weighted kappa (κ) values were calculated, and agreement was described according to Landis et al [18] as moderate, substantial, and almost perfect for κ values of 0.41–0.60, 0.61–0.80, and 0.81–1, respectively.

The endpoint OS was defined as time from operation to death of any cause or last follow-up. RFS was defined as time from operation to death of any cause or recurrence of CC. Patients later diagnosed with another cancer were censored at the date of diagnosis (*N* = 102). The median age was used as cut-off to dichotomize the parameter age. Survival curves were generated according to the Kaplan-Meier method and the log-rank test was used to test for differences between groups. The multivariable Cox-regression model was used to test for independent prognostic value with hazard ratio (HR) of 1.0 as reference and a 95% confidence interval (CI). A cut-off significance level of 0.10 was pre-specified for a variable to be included in the multivariable Cox regression model.

Chi^2^-statistics were used to test associations between clinicopathological variables. A *p*-value < 0.05 was considered significant. The statistical analyses were performed using STATA software version 14.0 (StataCorp, Texas, USA), and all statistical tests were two-sided.

## Results

### Patient characteristics

Patient characteristics are summarized in Table 1. In the follow-up period of seven years, 266 (46.5%) patients died; 110 (19.2%) patients experienced disease recurrence and 78 (13.6%) patients were diagnosed with another cancer. The median age at time of surgery was 73 years (range 29–95), and the mean follow-up time was 6.9 years (range 3–84 months).

**Table 1:** Clinico-pathological characteristics and association to PD-L1 (cut-off 5%)

	Number	PD-L1		
	(*N* = 572) (%)	Low (%)	High (%)	*p*-value
Age (years at diagnosis)				
Median	72			
Range	29–95			
≥ 73	267 (47)	253 (47)	14 (40)	0.414
< 73	305 (53)	284 (53)	21 (60)	
Gender				
Male	283 (49)	272 (51)	11 (31)	**0.028**
Female	289 (51)	265 (49)	24 (69)	
T-stage				
pT3	500 (87)	472 (88)	28 (80)	0.172
pT4	72 (13)	65 (12)	7 (20)	
Histology (WHO)				
Adenocarcinoma NOS	515 (90)	481 (90)	34 (97)	0.348
Mucinous adenocarcinoma	55 (10)	54 (10)	1 (3)	
Signet-ring cell carcinoma	2 (0)	2 (1)	0 (0)	
Malignancy grade				
Medium + low	450 (79)	436 (81)	14 (40)	**< 0.001**
High^a^	122 (21)	101 (19)	21 (60)	
Localisation				
Right	273 (48)	242 (45)	31 (89)	**< 0.001**
Left	299 (52)	295 (55)	4 (11)	
Tumour perforation				
Yes	530 (93)	499 (97)	31 (94)	0.313
No	17 (3)	15 (3)	2 (6)	
Unknown	25 (4)			
Lymph nodes				
Median	10			
Range	0–41			
< 12 nodes	351 (61)	329 (61)	22 (63)	0.851
≥ 12 nodes	221 (39)	208 (39)	13 (37)	
Perineural invasion				
Yes	26 (5)	25 (7)	1 (4)	0.635
No	359 (63)	337 (93)	22 (96)	
Not assessed	187 (32)			
Vascular invasion				
Yes	43 (7)	38 (9)	5 (22)	0.054
No	386 (68)	368 (91)	18 (78)	
Not assessed	143 (25)			
Mismatch repair status				
MSS	400 (70)	396 (74)	4 (11)	**< 0.001**
MSI	172 (30)	141 (26)	31 (89)	

Abbreviations: *MSI* microsatellite instability, *MSS* microsatellite stable, *NOS* not otherwise specified

^a^Including mucinous adenocarcinomas and signet-ring cell carcinomas

*P*-values are obtained using chi^2^-test. Statistically significant *p*-values are highlighted in bold

### PD-L1 expression

**Fig. 2:**
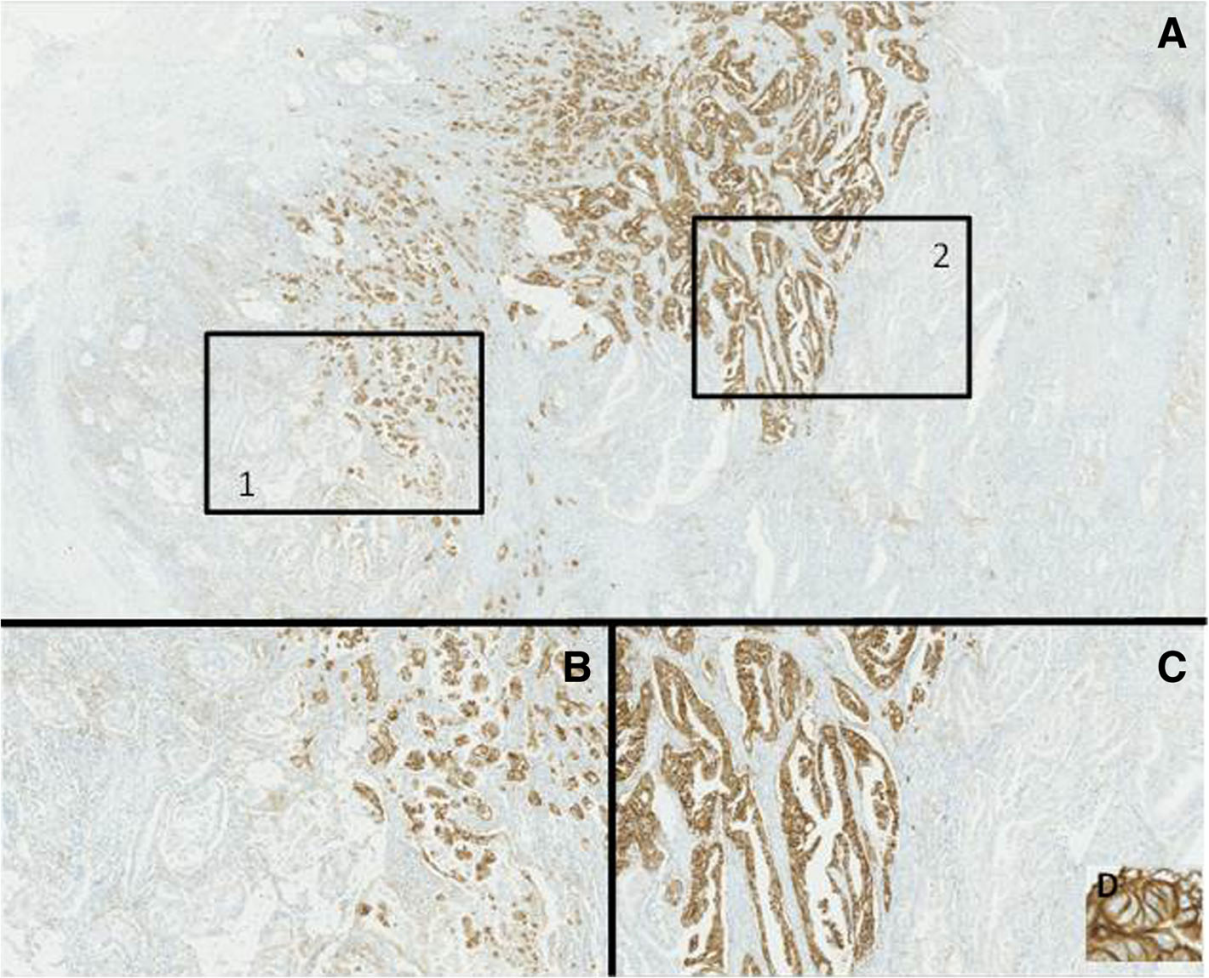
Example of heterogeneity of the expression of programmed death ligand-1. **a** Overview of the heterogenetic area. Frame one is presented in higher magnification in (**b**) and frame two in (**c**). The membranous expression pattern is displayed in (**d**)

The IHC staining of PD-L1 often had a highly heterogeneous expression both between the central part of the tumour and the invasive margin and along the invasive tumour front (Fig. 2).

Results regarding PD-L1 expression are displayed in Table 2. Nearly half of the population (46%) had no PD-L1 expression in tumour cells. After dichotomization, using 5% PD-L1 expression as cut-off, 35 (6%) of the tumours were classified as high PD-L1. In the group of MSI 31 (18%) of the tumours were classified as high PD-L1 and in the subgroup of MSS 4 (1%) were classified as high PD-L1. High PD-L1 was related to female gender (*p* = 0.028), high malignancy grade (< 0.001), right side localization (*p* < 0.001) and MSI (*p* < 0.001).

**Table 2:** Programmed death ligand-1 (PD-L1) expression in colon cancer

Number of PD-L1 positive tumour cells	Entire cohort (*N* = 572) (%)	MSI (*N* = 172) (%)	MSS (*N* = 400) (%)
None	264 (46)	47 (27)	217 (54)
0 < PD-L1 ≤ 1%	233 (41)	73 (42)	160 (40)
1% < PD-L1 ≤ 5%	40 (7)	21 (12)	19 (5)
< 5% PD-L1 ≤ 20%	10 (2)	8 (5)	2 (1)
< 20% PD-L1 ≤ 50%	11 (2)	10 (6)	1 (0)
PD-L1 > 50%	14 (2)	13 (8)	1 (0)

Abbreviations: *MSI* Microsatellite instability, *MSS* sMicrosatellite stable

The inter-observer agreement for the semi-quantitative evaluation of PD-L1 expression was moderate with κ = 0.418 and weighted κ = 0.573. The agreement improved to substantial, when categorizing the data into high PD-L1 expression (> 5%) or low PD-L1 expression (≤5%), κ = 0.691.

### Survival analysis

**Fig. 3:**
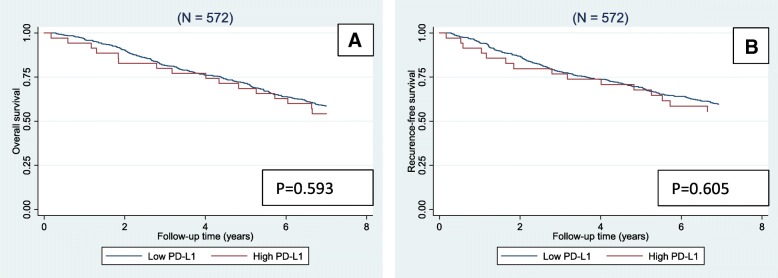
Kaplan-Meier survival curves depicting overall survival (**a**) and recurrence-free survival (**b**) stratified by the expression of programmed death ligand-1 (PD-L1) in the entire cohort (*N* = 572). *P*-values were calculated by log-rank test

**Fig. 4:**
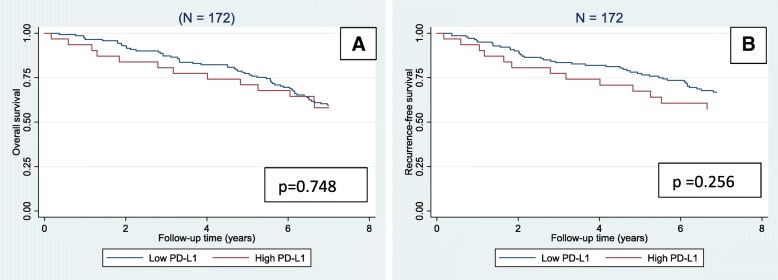
Kaplan-Meier survival curves depicting overall survival (**a**) and recurrence-free survival (**b**) stratified by the expression of programmed death ligand-1 (PD-L1) in the group of patients with MSI tumours (*N* = 172). *P*-values were calculated by log-rank test

The 5-year RFS for the population with low PD-L1 was 69.2% versus 67.7% in the group with high PD-L1 expression, and OS was 74.7% versus 70.5%, respectively. When considering patients with MSI tumours the 5 year RFS for low PD-L1 was 77.4% versus 67.5% in the group of high PD-L1, and OS was 79.4% versus 70.7%. No significant differences in survival rates were observed, considering the entire cohort (Fig. 3). In the group of patients with MSI tumours the Kaplan Meier curves were separated for RFS, but results were insignificant, *p* = 0.256 (Fig. 4). This also accounted the group of patients with MSI T3 tumours (*N* = 155), *p* = 0.149.

Outcomes from the corresponding univariable Cox regression analyses are shown in Table 3. Patients with MSI tumours and high PD-L1 expression did not have a significant worse OS or RFS, HR = 1.104 (0.604–2.016), *p* = 0.748 and HR = 1.429 (0.769–2.653), *p* = 0.258, respectively. Age ≥ 73 years, T4 tumour and perforation were significantly related to an adverse outcome of both OS and RFS. Patients with MSI T3 tumours and high PD-L1 expression did neither have a significant worse OS or RFS, HR = 1.531 (0.757–3.100), *p* = 0.236 and HR = 1.637 (0.833–3.216), *p* = 0.153, respectively.

**Table 3:** Univariable Cox regression analysis

Parameter	Overall survival		Recurrence-free survival	
	HR (95% CI)	*p*-value	HR (95% CI)	*p*-value
Age (years at diagnosis)				
< 73	1	**0.001**	1	**0.009**
≥73	2.533 (1.466–4.377)		2.194 (1.216–3.958)	
Gender				
Male	1	0.462	1	0.634
Female	0.839 (0.524–1.341)		0.881 (0.523–1.484)	
T-stage				
T3	1	**0.003**	1	**0.002**
T4	2.68 (1.409–5.128)		2.982 (1.503–5.915)	
Malignancy grade				
Medium/Low	1	0.536	1	0.636
High^a^	1.159 (0.726–1.852)		1.134 (0.675–1.906)	
Localisation				
Right	1	0.792	1	0.150
Left	1.084 (0.594–1.981)		1.576 (0.848–2.927)	
Tumour perforation				
No	1	**0.001**	1	**0.005**
Yes	5.332 (1.924–14.776)		5.644 (1.708–18.645)	
Lymph nodes				
< 12 nodes	1	0.440	1	0.220
≥12 nodes	1.203 (0.753–1.922)		1.386 (0.823–2.334)	
Perineural invasion				
No	1	0.630	1	0.873
Yes	1.417 (0.343–5.857)		0.850 (0.117–6.203)	
Vascular invasion				
No	1	0.878	1	0.756
Yes	0.922 (0.332–2.566)		0.830 (0.256–2.690)	
PD-L1				
Low	1	0.748	1	0.258
High	1.104 (0.604–2.016)		1.429 (0.769–2.653)	

Univariable Cox regression analysis regarding overall survival and recurrence-free survival for the sub-cohort of patients with MSI tumours (*N* = 172). Statistically significant *p*-values are highlighted in bold

^a^Including mucinous adenocarcinomas and signet-ring cell carcinomas

*MSI* microsatellite instability

Multivariable Cox regression analyses were not performed, as PD-L1 had *p*-value > 0.10 in the univariable Cox-regression analysis.

## Discussion

In this study, we investigated the prognostic value of PD-L1 expression on tumour cells in an unbiased, nationwide and population-based cohort of patients with stage II CC, treated exclusively with surgery. PD-L1 expression as a single marker did not provide any significant prognostic value regarding OS or RFS, neither in the the entire cohort nor in the subgroup of patients with MSI tumours.

In the entire cohort we found 6% of the tumours to have a high expression of PD-L1 on tumour cells, which is in accordance with other studies of CRC, reporting 5% positivity [11]. Likewise we found high PD-L1 expression associated to female gender, high malignancy grade, right sided localisation, and MSI, which also has been found by Lee et al, who investigated all stages of CRC [11]. Regarding MSI and MSS tumour subgroups, we found 18% of the MSI tumours to have high PD-L1 expression and 1% of the MSS tumours to have high PD-L1 expression. The difference in PD-L1 expression between MSI and MSS tumours has previously been descriebed in studies using a different scoring system [11, 19], although a recent study reported no differences in PD-L1 positivity among MSI and MSS tumours [20].

The association between MSI and high PD-L1 expression may be explained by the abundant infiltration of TILs in these tumours. Deficiency of the mismatch repair proteins results in a number of mutations. Therefore MSI tumours have a high load of tumour specific neo-antigens, which can induce an immunological response with recruitment and activation of T-cells [21]. One way to stimulate PD-L1 upregulation is afforded by the pro-inflammatory cytokine interferon-gamma (IFN-γ), which is produced by activated T-cells and Natural Killer cells [22]. The high expression of PD-L1 in MSI tumours with abundant infiltration of TILs is in accordance with the consensus molecular subtype (CMS) classification. The molecular group CMS1 is characterized by hypermutation, MSI and intense immune reaction [23], and this immunogenic group has been documented with a high PD-L1 expression [24].

In the group of patients with MSI tumours, the Kaplan-Meier curves were clearly separated regarding RFS, with a worse RFS related to a high PD-L1 expression, but statistical significance was not reached. This is in accordance with studies of tumour expression of PD-L1 in MSI stage I-IV CRC. Kim et al [9] reported a tendency towards a worse prognosis for tumours with high PD-L1 expression; however results were non-significant. Rosenbaum et al [10] reported no prognostic value for dichotomized data, but the group with the highest expression of PD-L1 (≥50%) had a markedly reduced disease-specific survival. We only found 14 patients to have PD-L1 expression ≥50%, and using this cut-off in our cohort did not enhance the prognostic impact (data not shown). Rosenbaum et al investigated all stages of CRC and found PD-L1 expression related to stage, which might explain the difference.

In contrast to our data, Koganemaru et al [12] reported high PD-L1 expression being an independent prognostic marker. They used the same cut-off (5%) in their evaluation of PD-L1, but they exclusively investigated stage III CRC. They found an association between PD-L1 and N status with high PD-L1 expression being related to higher N status. This may be part of the explanation for the inconsistency, as we only investigated stage II CC. Unfortunately, Koganemaru et al did not report any data on MSI status.

Lee et al [11] documented PD-L1 expression as an independent prognostic marker in patients with MSI tumours. In the present populationbased study 172 patients had a MSI tumour and only 31 (18%) of these tumours were classified as high PD-L1, resulting in a small group and thus low statistical power in the Cox regression analysis. This may be part of the explanation for the non-significant results.

Furthermore, previous studies reporting independent prognostic impact of PD-L1, differ in investigated cohorts and evaluation methods. Lee et al [11] included all stages of both colon and rectal cancers. Also their evaluation method differed in a number of ways from our technical approach. They used tissue microarrays (TMAs), as do most other studies [9–15]. In constrast we evaluated the PD-L1 expression in whole sections and observed a lot of heterogeneity both between the central part of the tumour and the invasive margin, and along the invasive tumour front. The use of TMAs may lead to selection bias, although several studies try to avoid this by using several representative TMAs from each tumour [9, 11, 13]. Lee et al [14] descreibe intra-tumoral heterogeneity of PD-L1 expression on tumour cells in 13% of the investigated tumours based on evaluation of TMAs from the central and invasive tumour compartments. They included all stages of CRC. In contrast we evaluated PD-L1 expression in a highly homogenous cohort of stage II CC patients and found a considerable intra-tumoral heterogeneity.

A standardized scoring system for PD-L1 expression in CC is missing and several unvalidated methods are in use. In the present study, the proportion of PD-L1 positive tumour cells was evaluated considering only membranous staining as positive. Intensity of the staining and cytoplasmic immunoreaction were not considered. Whether to take cytoplasmic staining of PD-L1 into account differs among studies. Some investigations use a combination of membranous staining and staining intensity [11] while others do not consider cytoplasmic staining at all [10]. PD-L1 expressed on the cell surface is essential for the interaction with the PD-1 receptor on the T-cells, indicating that only PD-L1 expressed on the membrane is of clinical importance. Furthermore, in the evaluation of lung cancer, only membrane staining is applied when evaluating the indication for immunotherapy with a PD-L1 inhibitor [25].

As mentioned above, we did not consider intensity of the staining. Intensity of IHC may be difficult to interpret. Poor reproducibility of IHC staining intensities of various proteins has been documented, while excellent inter-observer reproducibility was found estimating the fraction of positive tumour cells [26]. PD-L1 was not included in that study, but the same most likely applies to this protein. Furthermore, the evaluation of staining intensity is not only influenced by subjectivity. Various other factors may affect the staining intensity, encompassing both pre-analytical and analytical factors such as fixation (time and type), storage, and IHC protocols. Also section thickness affects staining intensity, and even modern state of the art microtomes produce sections with varying thicknesses.

The lack of a standardized IHC method challenges the assessment of PD-L1 expression, and moreover, different trials use different antibodies and assays. In melanoma, diverse assays have been found to variate in staining sensitivity of tumour cells [27]. For future studies, standardized techniques for evaluating PD-L1 in CC are required regarding antibodies, assays, interpretation, and threshold cut-off in scoring the immunostain. Also, focus should be directed on how to handle the marked heterogeneity, as this might hinder reproducibility of IHC scoring systems.

The present study is limited by the retrospective design, as we had no influence on the pre-analytical phase of the IHC. However, we used a validated antibody on a fully automatic platform, and only considered membranous staining according to the manufactures recommendation. The PD-L1 antibody used in this study stained both the malignant epithelial cells and immune cells in the stroma, which made it difficult to discriminate these cellular populations when the density of immune cells in the tumour stroma interface was high.

The prognostic value of PD-L1 expression on tumour cells is controversial. We investigated the expression of PD-L1 exclusively in stage II CC in a well-defined and unbiased population, and did not find any prognostic value of PD-L1 as a single biomarker. The PD-L1 expression in tumour cells should be seen in the context of the entire immune tumour microenvironment. The expression of PD-L1 is influenced by TILs, which is related to MSI. We did not prove any prognostic value of PD-L1 in patients with MSI tumours, however this interaction should be taken into consideration in future studies.

## Conclusions

In this nationwide population-based cohort of stage II colon cancer, we found membranous PD-L1 expression (cut-off 5%) in tumour cells of stage II colon cancer to be associated with female gender, high malignancy grade, right side localisation and MSI. The expression of PD-L1 was often highly heterogenous. No prognostic information was detected of PD-L1 as a single biomarker in this cohort of stage II colon cancer.

## Abbreviations

CC: Colon cancer
CRC: Colorectal cancer
DCCG: Danish Colorectal Cancer Group
HR: Hazard ratio; IHC: Immunohistochemical
MSI: Microsatellite instability
MSS: Microsatellite stability
NOS: Not otherwise specified
OS: Overall survival
PD-1: Programmed death receptor 1
PD-L1: Programmed death ligand 1
RFS: Recurrence-free survival
TILs: Tumour infiltrating lymphocytes
TMA: Tissue microarray
